# Having a Child Diagnosed with Cancer: Raising the Challenges Encountered by the Caregivers at the Pediatric Oncology Ward in Egypt

**DOI:** 10.3390/diseases5040036

**Published:** 2017-12-19

**Authors:** Hanan El Malla

**Affiliations:** 1 Department of Social Work, The University of Gothenburg, 405 30 Gothenburg, Sweden; hanan.el.malla@socwork.gu.se; Tel.: +46-735-004-512; 2 Department of Clinical Cancer Epidemiology, Sahlgrenska Academy, The University of Gothenburg, 405 30 Gothenburg, Sweden

**Keywords:** cancer disease, pediatric oncology, Egypt, culture, caregiver, psycho-oncology, psychosocial oncology

## Abstract

Having a child diagnosed with a life-threatening illness, and undergoing a severe treatment regimen, is a massive challenge for many caregivers, not the least of who are those with low socioeconomic status and living in a society where deeply rooted cultural and societal misconceptions are immensely noticeable. The aim of the study is to raise the great concerns experienced by the caregivers at the pediatric oncology ward in Egypt. The study is comprised of 24 caregivers of children with cancer undergoing treatment at the inpatient ward. Semi-structured interviews and participant observations were used as a means of data collection. Numerous concerns are addressed in this study which are all related to fear of the illness and guilty feelings of having caused the child this illness. The fears and concerns addressed in this paper seem to obstruct the caregivers’ overall psychosocial wellbeing, which is known to have multiple effects on the child’s overall wellbeing. Thus, it is very important to take into consideration caregivers in the child’s cancer treatment.

## 1. Introduction

Despite the medical progress in the field of pediatric oncology, a gap between the medical and psychosocial fields is evident, especially for an underdeveloped country like Egypt, which is the most populous country in the Arab world and third most populous country in Africa [1]. Egypt has a population of 97 million, and 95% of the population live condensed in only 5% of the land area, along the Nile River [1]. The overall literacy rate in Egypt is 72%, and 25% of the population is living below the poverty line [1]. The high incidence rate and prevalence of childhood cancers in the region is alarming, and the survival rate is between 20% and 25% [2], which is very low compared to the US and Sweden where the rates are between 85% and 95%, depending on the specific tumor [2]. The medical efforts are visible, yet, very little is known of the psychosocial experiences of the caregivers who carry an extraordinary burden and play a significant role for the child. Thus, recognizing the thoughts, needs, and concerns of the caregivers is very important as it reflects on the child’s emotional and physical well-being, which affects adherence to medication, disclosure of information, and even survival [3,4,5,6]. To our knowledge, there are very few published studies in international journals addressing the concerns of caregivers of sick children with cancer in Egypt. Thus, the aim of this study is to provide an account of the caregivers’ challenging journey at the oncology ward and contribute to raising the unheard voices speaking of their concerns and fears. The study was conducted at the major governmental hospitals in Cairo, Egypt.

## 2. Materials and Methods

### 2.1. Study Population

The study population was comprised of *n* = 24 caregivers to children with cancer at the oncology ward. All caregivers attending the outpatient cancer clinic whose child would be admitted for treatment at the inpatient ward were approached, however, only those who fulfilled the criteria were asked to participate. Having a child between the age of 9 to 15 admitted to the inpatient clinic for treatment was the inclusion criteria in this study. Children below the age of 9 were excluded due to ethical issues in interviewing a child at this young age.

The study was conducted at Cairo University Hospital which is Egypt’s main governmental university hospital, and the interviews were conducted during a 5-month period in 2005. PI El Malla approached the informants fulfilling the above criteria upon admission. The caregiver/s were first informed by the responsible oncologist that PI was conducting a study, and the caregiver/s were asked if they would like to participate. If the caregiver/s gave consent to participate, a brief introduction to the study and its aim was provided. Together with the PI, an arrangement for the time of the interview was made. None of the approached caregivers refused participation in the study. The Research Ethics Committee in Cairo, Egypt approved the study.

Based on this study, a large-scale prospective study was conducted in 2008 and was followed up in 2013 [3,4,5,6] including two comprehensive, structured interview questionnaires with 304 caregivers to newly diagnosed children with cancer starting their first chemotherapy treatment, followed again by the third chemotherapy treatment, and finally surveyed five years later as a mean of a five-years survival rate follow-up [6]. The study was conducted at the Children’s Cancer Hospital in Egypt (CCHE), the largest pediatric oncology hospital in the region. Despite the fact that CCHE is a large hospital and has been operating since 2007, the hospital is not able to accommodate all pediatric oncology cases.

### 2.2. Data Collection

The study preparation was conducted at the National Cancer Institute (NCI) in Cairo, Egypt and included caregivers (*n* = 5). Thereafter, to assure validity and reliability, in other words, to ensure that all questions were fully understood the way we intended and measured what we were looking to measure, face-to-face validation was conducted (*n* = 4). The final interview guide was validated in a pilot study including *n* = 18 caregivers.

The data collection for this study consisted of a structured, in-depth interview guide, entailing 40 questions, as well as participant observations which played an important role in the understanding of the interviews and the interaction at the ward.

The interview guide was translated from English into colloquial Egyptian Arabic and back-translated, and all formal interviews were audiotaped and transcribed into English. PI, who is bilingual, knowing both Arabic and English, conducted all translations and interviews throughout the study.

### 2.3. Interviews

The interviews were commenced by informing the caregiver/s that their participation was voluntary and that they could discontinue the interview at any time or avoid answering any of the interview questions without any further explanation. They were also informed that their identities would remain completely undisclosed to ensure confidentiality and anonymity, and their non-participation, participation, or discontinuation of the interview would not have any consequences on their child’s treatment process. After a brief introduction of the PI and the institution that this study was conducted under, the PI introduced the aim of the study and asked for permission to audiotape the interviews.

The interviews were audiotaped and transcribed to English as they were conducted in Arabic. The transcriptions are not detailed in the sense that non-linguistic features were not included. Furthermore, some of the translations of metaphors or domestic common sayings were translated into the context of the English language, which entails the PI’s understanding of it.

### 2.4. Methodological Approach

For the purpose of this paper, a social constructionist approach was adopted, since it was intended to uncover the social rather than the individual roots of problems; such understanding will provide us with an objective understanding of how to consider the social construction of explanations to problems [7] which is the core of this paper. With the use of the sociological “eye” we will learn to think critically about the “common wisdom” surrounding the different areas [8], such as death and incurability. When considering a social problem, we need to study the process that creates it, which is very important for a holistic perspective and as a mean of prevention, since the caregivers in this study have a major influence on their children’s lives. Social constructionist researchers in psychology have acknowledged the importance of focusing on psychological aspects, such as emotions [9], and this is important as it illustrates how this approach is concerned with human feelings, thoughts, and ideas and is not a mere description of a certain condition, situation, or setting.

Interpretive phenomenological approach (IPA) was adopted as a qualitative research method of data collection commonly used in critical health psychology. The IPA method is developed to provide a closer look at the informant’s psychological world in relation to social and cultural contexts [9]. IPA is concerned with health psychology as it presumes a link between cognition, physical state, and verbal response [10]. Since the research is dealing with the beliefs and thoughts of the caregivers at the oncology ward, this method was used in order to understand how the caregivers view and experience life, their world, their child’s illness, and the hospital stay [9]. The method does not question the individual’s thoughts, ideas, and view of the world as true or false, rather, it aims at finding the informant’s experience of the environment around them [9]. Nevertheless, it also acknowledges the fact that an informant’s experiences, thoughts, and feelings about things in life develop through his or her interaction with the social surrounding [9]. IPA method has a benefit in constructing a cognitive “map” for the researcher that includes the informants’ view of their situation [9]. Some analytical techniques have been used from the Grounded Theory as it is very closely related to IPA. Grounded theory has broad data collection options in terms of transcription of audiotaped semi-structured interviews, participant observations, memo writing, and making use of a “focus group” [9]. Hence, both approaches are similar to each other as both focus on “themes and categories” that appear through analysis [9].

### 2.5. Data Analyses

There was no data analysis software applied in this study. The data was extensively and thoroughly read, analyzed, and categorized into codes. The codes were later clustered into groups and then categorized again to detect the most recurring themes brought forward by the informants. As a final step, one main recurring theme was identified and further analyzed, categorized, coded, and addressed in this paper. The data included many themes, which are not all addressed here due to the specific scope and aim of this paper.

## 3. Results

This paper included interviews with a total of *n* = 24 caregivers of children diagnosed with cancer staying at the inpatient clinic at Cairo University Hospital (Table 1). This paper brings forward the caregivers’ accounts about their child’s disease, which is a key theme in the data provided. The caregivers’ narratives regarding their child’s disease are divided into two main psychosocial streams; (1) misconceptions associated with eminent guilt feelings towards the child in the assumption of having caused the child the illness through numerous malpractices and (2) misconceptions arising from the horrendous fear of the illness and the stigma attached to it.

### 3.1. Feelings of Eminent Guilt Towards the Child

A stream of concern addressed by many caregivers in this study was the perseverant feeling of guilt associated with the etiology of the child’s disease. When the caregivers are told at the hospital that the etiology of their child’s illness is unknown, there seems to be an involuntarily and unconscious, deeply rooted cultural assumption that there must be environmental factors involved, as the societal medical discourse is based on reasoning where diseases ought to have an origin, which is true for many diseases, yet not all. The malpractices referred to by the caregivers can be divided into three streams: (1) lack of hygiene and hazardous exposure; such as (a) neglect of the child’s personal hygiene and (b) exposing the child to polluting and hazardous environments, (2) having provided the child with unhealthy dietary products or/and not attending to the child’s various intake, as several parents shamefully disclosed that their child had on an occasion or two ingested its own feces during toilet visits, and (3) stress during pregnancy.

### 3.2. Fear of the Illness and the Misconceptions and Stigma Attached to It

Seven recurring worries and fears were identified: (1) fear of naming the illness as it may bring a bad omen and infect the person who mentions it, (2) fear of the uncertainty of whether the illness can be transmitted or not to siblings, peers, neighbors, and other family members and whether it is a heavenly curse or not, (3) fear of whether the illness has a genetic disposition, (4) fear of the side effects of treatment such as loss of hair, eyebrows, fertility, vision to one or both eyes, hands, or feet, (5) fear of losing the main source of income due to the unexpected expenses brought about a prolonged treatment regimen, (6) fear of being/becoming a burden to the family by being absent for long periods of time, and (7) fear of the death of the child.

## 4. Discussion

Among the 24 caregivers interviewed in this study, two main psychosocial streams were identified as major challenges for the caregivers: (1) misconceptions associated with eminent guilt feelings towards the child in the assumption of having caused the child the illness through numerous malpractices and (2) misconceptions arising from the horrendous fear of the illness and the stigma attached to it.

According to research, cancers occur through an interaction between genetic and environmental factors [11]. However, between 75% and 90% of childhood cancers are poorly understood, and the causes are unidentified [12,13]. This is the information most caregivers receive at the ward. The caregivers interviewed in this study disclosed a vast frustration and disbelief in regards to the information provided at the hospital of the unknown etiology of their child’s illness. Hence, the caregivers construct and live with the conviction that certain malpractices they are responsible for caused the occurrence of their child’s illness. The three main streams of malpractices addressed by the caregivers include: (1) lack of hygiene and hazardous exposure, (2) unhealthy diet, and (3) stress during pregnancy. These are further re-produced among the caregivers during their stay at the ward and originate to a large extent from deeply rooted misconceptions in the society.

Mindful hygiene and avoidance of hazardous exposure is common advice very eagerly given by physicians as they may have an effect on the immune system and bring about unfavorable complications and prolong the treatment regimen which in turn may have life threatening outcomes. The dilemma for most of these parents in this socioeconomic class is that the main available outlet for their children is usually those places that the child may be exposed to pollution from cars, street garbage, or any sharp items with which the child may come in contact. For many children, the community in the street becomes like a second home, where hours of play are spent as a mean of escaping a home where domestic violence, constant arguments, disputes, or emotional abuse might be happening. Hence, the street is usually a source of entertainment for the children and a getaway from the difficult home situation. Also, seldom are there football pitches or playgrounds available for children to spend their time at, and, where they are available, they are usually in the middle of the city with high levels of pollution from cars. In other words, these caregivers can rarely afford a safe place for their children to be, which brings about additional agony and feelings of guilt and failure. Also, many of these families live in such harsh conditions that they do not have access to clean water or even a safe roof.

The consequences of the difficult socioeconomic situation and the very few resources provided to caregivers can be seen as a consequence of some of the practices they address. Hence, exposure to a hazardous and contaminated environment is evident indoors as well as outdoors for many of these children. Clean water and sewage water are not available resources for millions of people in Egypt, which could bring about various unintentional malpractices. In the cases where water is provided, it is expensive and/or scarce, which is an additional constraint for the caregivers as they need to be aware of their water consumption and hygiene becomes, naturally, the last priority. Also, in many cases, there is a lack of availability of water, and those households shower out in ponds or along the Nile River which is certainly not recommended as these are usually contaminated. Additionally, for many of these children, living in poor, crowded, and improper homes, the only outlet they can afford is outdoor play which has its hazards in an overpopulated and underdeveloped country like Egypt; it is either overpopulated with excessive exposure to carcinogenic automobile fuel, or it can be out in the farms where the use of carcinogenic sprays is a very common practice among farmers.

Moreover, the dietary awareness and, thus, habits among this group is seemingly unbalanced due to financial constraints and lack of awareness to a certain extent. This is an increasing worry for many caregivers during their child’s treatment, as the physicians constantly remind them to look after their child’s dietary habits, make sure the child is well fed, and is restricted from certain foods. This is another vicious cycle the caregivers are finding themselves trapped in as the means for a healthy diet are not easily attained. In addition, the excess intake of carbohydrates (rice and potato as they are cheap/subsidized and available throughout the year) brings about an unbalanced diet with very little portions of protein, vegetables, and fruits; however, it does silence a hungry stomach. Hence, undernourishment, the excess use of affordable yet unhealthy products, unhealthy cooking, such as excessive frying of potato, and the use of unclean water are major challenges for many households in Egypt, whether in urban or rural areas. Sadly, the challenging socioeconomic situation of the caregivers has become an obstacle for the children and seems to put them at risk for developing numerous diseases and stagnating or even prolonging the treatment process of the diagnosed child.

Furthermore, the socially and culturally constructed blame directed towards the mother is evident in this study, as many caregivers/mothers in this study were blamed for causing their child’s illness by being stressed during pregnancy and, therefore, held responsible to some extent. This seems to bring about an increased worry and an excessive feeling of guilt, great disappointment, and self-doubt which asserts the mother’s negative self-image as an inappropriate maternal figure that, through acts of ‘foolishness’ as addressed by several caregivers, have caused the child a fatal disease. This is narrated by many mothers in this study and, sadly, it is not only confirmed and validated by the surrounding network of the caregivers (oftentimes the mother, mother-in-law, and in some instances the husbands), it is also fostered and fed immensely at the ward among the caregivers, consequently becoming a truth as observed on numerous occasions.

All caregivers in the study addressed elevated worry since their child’s diagnosis. The accumulation of fears seems to be never-ending for some caregivers as their tremendous fears are related to their child’s illness, and these fears are clearly extremely challenging for them to contain and acknowledge. The fears and concerns addressed by the caregivers as mentioned above can be understood and analyzed in various ways: 

(1) Fear of naming the illness as it may bring a bad omen and infect the person who mentioned it. The stigma and misconception around the illness brings fear to many people and the media has associated the illness with various dramatic and deadly outcomes. This has brought about an immense fear of the illness. Many caregivers do not feel comfortable when the word “cancer” is used and when PI would use it, the immediate answer was “god forbid”, and, hence, the most commonly used term for cancer was “the malicious, evil disease”, which in Arabic is a very negative word to use and entails suffering and evilness. This explains, to some extent, why the caregivers exhibit a horrendous feeling when they receive the diagnosis which could be comparable to receiving a death sentence of one’s child. This phenomenon is addressed in a study by El Malla et al. which found the caregivers refrained from, and deliberately avoided, using the word cancer [5]. It is also addressed in several other studies on adult cancer patients in this region such as the study in Saudi Arabia where family members desired more information but clearly discouraged disclosure and information provision to the adult patients [14,15,16,17,18] or Pakistan where similar patterns were found among adult cancer patients and their family members [19].

(2) Fear of uncertainty as to whether the illness can be transmitted or not to siblings, peers, neighbors and other family members, and whether it is a heavenly curse or not. It is interesting that none of the caregivers would explicitly address their fear of having the disease transmitted to them as it seemed to be an act of selfishness in this societal context. When PI addressed this issue, the caregiver/s would reply “it is all in the hand of the ‘creator’”. Many caregivers address the stigma they face when, for instance, their neighborhood finds out of their child’s illness and family members distance themselves fearing contagion of the illness or the bad reputation a malignant disease might have in this cultural context. Another important worry addressed is the loss of one’s status in the extended family/neighborhood or tribe which can be related to being “cursed” and thus excluded and deprecated.

Many of the caregivers have a strong belief that their child’s illness is a curse from the creator due to their misbehavior, malpractice of religious rituals, or way of life; while a few other caregivers perceive it as a positive test from the creator. In other words, it was viewed as either a test due to malpractices or a voice of call to ‘return’ to the creator. Interestingly, several caregivers expressed that they did not plan their pregnancy and when they found out about it, they were very upset and wanted to undergo abortion, and they feel this is an explanation for being cursed and having to go through this illness with the child. This mindset has no religious background and is socially and culturally constructed. A study conducted by El Malla et al. addressed the caregiver’s need for information. The study showed an association between information provision and a higher level of trust in health-care professionals [3]. The study also found an association between the mother’s level of education and the child’s higher survival in a 5-year follow up [6], which emphasizes the need for education and information provision.

(3) Fear of whether the illness has a genetic disposition, which means that, in the case where the child has a sibling or the mother is expecting or planning on conceiving another child, the siblings could develop the disease. This could mean the loss of a child and also, a repetition of this “everlasting journey”. This fear fosters another concern which is that of being isolated and stigmatized by the extended family members, neighbors, school peers and teachers. Many caregivers ask the oncologists about the probability of having the disease transmitted to siblings and the reply is in line with “it is all in the hands of the creator” which could mean yes or no, although this is not a vague answer for many of the caregivers who expressed their tendency to believe it to mean yes. This uncertainty creates a terrible fear which is fostered and mourned at the ward among the caregivers.

(4) Fear of the side effects of some treatment regimens such as loss of hair, eyebrows, fertility, vision to one or both eyes, hands, or feet. Hair loss is an issue caregivers are concerned with, not only due to the change of appearance the child may experience which can be very difficult, but in some instances, it seems to be more of a problem for the caregiver than the child. The hair loss brings up many questions at school and in the neighborhood and can be problematic if the caregivers have decided not to disclose the child’s illness to their community. Also, for girls in particular, losing one’s hair is similar to a loss of one’s gender identity which is important in the society’s view of how girls should look. The hair loss brings the fear of additional losses, as addressed by many caregivers, in a time of countless losses such as bodily and emotional changes, as addressed by El Malla [20] (article in press) and Usmani [2] as well as another study conducted in Egypt by Fawzi et al. where physical appearance was addressed as an obstacle and correlated with lower quality of life for the children [21].

Additionally, and most importantly, is the significant and irreversible loss of fertility which many caregivers sadly grieve severely. The possibility of such loss brings about societal impediments and impediments for the child’s future; nonetheless, it seems to be more of an issue for the females as among certain families their self-worth is to some extent associated with the ability to conceive; reproduction and family expansion is thought to be the responsibility of the woman. This issue seems to be one of the most critical yet silent fears. The societal and cultural misconceptions become close and too difficult to contain and this where cognitive dissonance is apparent. The caregivers struggle in their minds and thoughts as to whether this is a valid perception, and it becomes an almost impossible struggle to combat. Furthermore, this fear feeds a horrendous thought that the society will not accept one’s child for marriage, even if the child is cured, due to the inability to conceive or a history of carrying a highly heredity gene of cancer.

This chain of immense concerns is fed by the additional assumption that even siblings will not be able to marry as society assumes that cancer is transmitted or is genetically inherited which means that, even if the sibling does not develop the disease, he/she will not be socially accepted for marriage and might be deprecated and stigmatized by society. The concerns shift from the child’s illness to the entire family’s future as well as their worth in society, community, and life as a whole. For the caregivers, the scenarios become gloomier and more difficult to contain, growing in proportion and intensity. As quoted by several caregivers, sadly, it feels like “*an everlasting journey*”, “*a broken chain; unrepairable and therefore invaluable*”. The socially constructed view of a human being having value solely for her ability to conceive is very difficult for caregivers to combat as well as tolerate, yet it is the truth for many. Suddenly, one’s child is no longer seen as a member of the tribe but as a “*deviant outsider*” as addressed by numerous caregivers in this study when the infertility subject was brought up.

(5) Fear of losing the main source of income due to the unexpected expenses of a prolonged treatment regimen. Due to the nature of the illness, many parents have lost their source of income, and some others have lost their jobs as employers do not accept prolonged and unexpected emergency absences. The society does not have a support system to compensate the caregivers’ absences from work, which makes them very fragile to the economic situation the illness brings about. Women in Egypt represent a major working force, and many of the caregivers in our study were very concerned with the economic burden they were experiencing. For instance, PI observed a recurrent delay in following the treatment, and during observation and numerous informal interviews, caregivers would disclose that they were late to treatment due to financial constraints, as well as lack of trust in the treatment regimen, which was also addressed in a study by El Malla et al. [3]. The financial constrains generate a barrier for many of the caregivers whom were late as some could not leave work or had to stay for additional days fearing the loss of their job due to frequent absences. Also, some could not afford to pay for transportation to the hospital as it could take several hours and require several kinds of transportations (train from Upper Egypt to Cairo may take up to 12–15/20 h and then riding a microbus to the city center and another microbus to the hospital), and these long journeys cost a relatively large sum of money that many families struggle to earn. This barrier is also addressed by Usmani along with numerous barriers [2]. The economical constrains are also addressed by Fawzi et al. which have shown to correlate to lower quality of life among the children [21].

(6) Fear of becoming a burden to the family by being absent during long periods of time. Since many caregivers have other children, the extended family becomes an important pillar to uphold the family structure. Again, due to prolonged periods of treatment, the nature of the treatment, and the emergency treatments in-between, many caregiver’s relation with extended family members is put under pressure, and frictions tend to arise as the burden increases. Additionally, some caregivers were concerned that the members of their extended family might renounce the responsibility of the children as the burden grows, which might be devastating for some caregivers whose network will not bear this kind of serious responsibility. Additionally, caregivers are also concerned that family members might not be suitable or appropriate to care for the children, which adds to the frustration and anxiety experienced and brings about guilt feelings towards the children left at home. Several caregivers were exchanging various terrible narratives of suspected physical and emotional abuse and neglect of their children during their absence. In a study conducted by Fawzi et al., a significant lower quality of life was observed in children who spent more than two thirds of their illness at the hospital [21]. This could indeed relate to the caregiver’s immense challenges at the hospital which then affect the child.

(7) Fear of losing the child. Death is undoubtedly a main concern for many caregivers. This is to some extent due to the high mortality rate in the region; 75% of cancer diagnoses in children result in death [2]. Additionally, as addressed above, for several caregivers, the death sentence was issued “when the physician uttered the name of the illness” and for others it is “when told that the child might lose fertility as a side effect of the treatment” as quoted by many caregivers. The latter is shocking but not unusual for a society where fertility is seen as part of a human being’s survival, and to a large extent, societal worth, and status. Thus, many caregivers are very concerned and they collectively mourn their own and their children’s losses, and fear the death of their child which is also addressed in a study by Grootenhuis et al. [22]. This is illustrated through the numerous observations at the ward during the night time when the children are asleep and the caregivers gather in the corridor and collectively share information, the burden, and the stories of sadness, worries, and the fears and concerns of what is yet to come.

Having a child diagnosed with a life-threatening illness in an under-developing country like Egypt is devastating for many caregivers, including the caregivers in our study. The economic situation, the cultural and societal pressure and misconceptions, as well as the illiteracy among our study group is extremely challenging for the caregivers. Also, staying at the hospital for long periods of time and on a frequent basis seems to tear apart the relationship between the caregiver and his/her social network, especially for those living miles away from the hospital. Thus, the caregivers are often alone when they are not with the other caregivers at the ward, making mention that they cannot handle the loneliness and expressing their need to share their thoughts and concerns with other caregivers in their same situation is vital. As a consequence, the loneliness and unreliable sources of information seem to be creating a sense of group affiliation, serving the need of community belonging and the urgency for collective grieving, however, it seems also to be feeding deeply rooted cultural and societal assumptions and misconceptions that have been observed to have very tough consequences on the wellbeing of the caregiver and consequently the child.

Interestingly, though the study addressed in this paper was conducted in 2005, the above-mentioned studies conducted from 2008 to 2013 by El Malla et al. as well as other studies conducted by Fawzy et al. in 2013 [21], Al-Amri in 2009–2011 [14,15,16], Aljubran in 2010 [17], and Jawaid in 2010 [18] all address very similar, if not identical, patterns of thoughts, ideas, and concerns as those addressed in this paper. This might be an indication of the slow progress and sometimes stagnant pace of the psychosocial field of oncology in Egypt and the slow progress of breaking the cultural misconceptions. It also brings forward the need for further studies in this area as well as psycho-educational interventions. A strength in this study is the bilingual competence of the PI which facilitated the collection of the data and the analysis as there was no need for translation and the data was handled within its cultural context without any difficulties in understanding the meaning of the data [23]. Due to the specific purpose of this paper, the voices of the children and physicians are not present, which is a limitation, nonetheless, a paper in progress [20] and additional future papers will be provided to fill this gap. Additionally, to ensure the confidentiality and anonymity of the study and in order to be able to conduct the interview with no disruption, the PI had to stay at the ward for long hours and attend many night shifts. This was due to the partial unavailability of the conference room. Thus, some of the interviews were conducted after the regular working hours. Nevertheless, this brought about a more personal relationship between the PI and the informants and the health-care professionals at the ward as it provided the PI with an extensive and intensified insight of the situation and enabled many significant informal dialogues.

## 5. Conclusions

The majority of the caregivers in this study are illiterate and their main source of information, according to them, is the word of mouth of neighbors, relatives, colleagues at work, and to a large extent, the surrounding caregivers at the ward. Access to information through reliable sources such as books or reliable medical websites as well as social media and other modes of information provision is very difficult for this group of caregivers whose literacy level is very low. The illiteracy goes together with the ignorance regarding the various cancer diseases and their etiology. People do not have access or know how to access statistical data. In addition there have been decades of enforcement in the society portraying cancer as one single disease with only one disastrous outcome: a painful, prolonged, tragic, and un-escapable destiny: death. Along with that, as the cancer prevalence and incidence rate is high and increasing in Egypt, there has been a growing public concern, in particular, when various hospitals in the media addressed the disease as a major public health burden and encouraged the citizens to donate sums of money. Another factor contributing to the ignorance is the mode of language used for the majority of the reliable websites, as English is the main language of communication. So, even if the person is literate, browsing through the internet for reliable sources and finding reliable information in Arabic is a major hindrance for many people, and, consequently, they end up resorting to the familiar media forums which are usually the TV or radio and the word of mouth of neighbors, relatives, colleagues at work, and the other parents at the ward.

The clinical implication of this paper was to make the voices of the caregivers of children with cancer heard. That is, to encourage a better understanding of the suffering and endless losses in a very prolonged, painful, and persistent journey. The paper addressed cultural, societal, as well as religious misbeliefs, since they are very important to be considered in the clinical setting. The fears and concerns addressed in this paper seem to obstruct the caregivers’ overall psychosocial wellbeing, which we know has multiple effects on the child. Moreover, these data are equally important to health-care professionals working with this ethnic group in the West as the enormous immigration flow from this part of the world is at its peak. Cultural misconceptions are sometimes very specific and not by default comprehensive for other cultures, and they are sometimes rigid and hence very difficult to modify and challenge. Hence, the data in this article are important for physicians, nurses, social workers, psychologists, physiotherapists, and other health-care professionals working with these children and their caregivers on a daily basis and for prolonged periods of time. Acknowledging these cultural misconceptions and addressing them in the clinical setting could be a first step in breaking the vicious cycle of misconceptions and establishing a rapport with the child and his/her caregivers with less presumptions and more focus on the child and the caregiver.

## Figures and Tables

**Table 1:** Basic characteristics of the caregivers and their children.

ID of Child	Age of Child/Years Old	Sex of Child	Diagnosis of Child	Caregiver/s Interviewed
1	10	M	ALL	Mother, father
2	10	F	ALL	Mother, father
3	10	M	ALL	Mother
4	10	M	ALL	Mother, father
5	11	F	AML	Mother, father, sister
6	15	F	Osteo. Sarcoma	Mother, grandmother
7	15	F	AML	Mother
8	15	F	AML	Mother, sister
9	10	M	ALL	Mother, brother
10	15	F	Osteo. Sarcoma	Mother
11	15	F	Osteo. Sarcoma	Mother
12	13	M	PNET	Mother, father
13	11	F	AML	Mother, father
14	11	F	Hodgkin’s Disease	Mother, father
15	9	F	PNET	Mother, grandmother
16	14	F	Brain Tumor	Mother, father
17	9	F	Willms Tumor	Mother
18	12	M	PNET	Mother, grandmother
19	9	M	ALL	Mother
20	12	M	Osteo. Sarcoma	Mother, grandmother
21	13	F	Hodgkin’s Disease	Mother, sister
22	14	M	ALL	Mother, father
23	9	F	ALL	Mother, grandmother
24	12	M	AML	Mother, grandmother

AML; acute Myeloid leukemia, ALL; Acute Lymphoblastic Leukemia, PNET; Primitive Neuroectodermal Tumors
